# Dietary consumption of selenium inversely associated with osteoporosis in postmenopausal women

**DOI:** 10.3389/fnut.2022.997414

**Published:** 2022-09-12

**Authors:** Patricia Paula da Fonseca Grili, Camila Vilarinho Vidigal, Geise Ferreira da Cruz, Ben Hur Albergaria, José Luiz Marques-Rocha, Taísa Sabrina Silva Pereira, Valdete Regina Guandalini

**Affiliations:** ^1^Postgraduate Program in Nutrition and Health, Health Sciences Center, Federal University of Espírito Santo, Vitória, Espírito Santo, Brazil; ^2^Department of Integrated Education, Health Sciences Center, Federal University of Espírito Santo, Vitória, Espírito Santo, Brazil; ^3^Department of Social Medicine, Federal University of Espirito Santo, Vitória, Espirito Santo, Brazil; ^4^Department of Health Science, University of the Americas Puebla, Cholula, Mexico

**Keywords:** menopause, osteopenia, nutrients, dietary intake, bone mineral density

## Abstract

**Background:**

Osteoporosis is a skeletal disease characterized by reduced bone mineral density (BMD), which increases the risk of falls and fractures and reduces mobility. Some nutrients have a well-established role in maintaining bone health and preventing osteoporosis, while selenium (Se) has aroused interest in bone health possibly because of its anti-inflammatory and antioxidant capacity. The aim of this study was to evaluate the association between dietary Se consumption and BMD in postmenopausal women.

**Materials and methods:**

Cross-sectional, observational, analytical study carried out with women in menopause for at least 12 months, aged ≥ 50 years. Sociodemographic, lifestyle, and clinical data variables were studied. BMD was assessed using Dual Energy X-ray Absorptiometry (DXA) and the participants classified as having normal BMD, osteopenia, or osteoporosis. Dietary consumption of Se was assessed by the food frequency questionnaire (FFQ) and classified into quartiles of consumption. Multivariate logistic regression with three fit models was applied to investigate the association of BMD with Se consumption quartiles. The significance level adopted for all tests was 5.0%.

**Results:**

The final sample consisted of 124 women aged in average 66.8 ± 6.1 years and with a time since menopause of 19.6 ± 8.8 years. According to the BMD, 41.9% of the women had osteopenia and 36.3% osteoporosis. The mean consumption of Se was 154.4 ± 88.7 μg/day. The highest consumption of Se was observed among women with normal BMD (51.9%), whereas lower consumption levels were found in 57.7% of women with osteopenia and in 60.0% of women with osteoporosis (*p* = 0.003). In the multivariate analysis, after adjusting for possible confounding variables, Se remained associated with the group of women with osteoporosis. Postmenopausal women in the highest quartile (≥94.0 μg/day) of Se consumption had an OR of 0.02 (95%CI: 0.001–0.41; *p* = 0.012) of having osteoporosis when compared with women in the lowest quartile.

**Conclusion:**

Se consumption was associated with BMD and postmenopausal women with higher Se consumption were less likely to have osteoporosis.

## Introduction

Osteoporosis is a skeletal disease characterized by reduced bone mineral density (BMD) and deterioration of the bone tissue microarchitecture, with a consequent increase in the risk of falls, fractures, and reduced mobility (1, 2). In a meta-analysis carried out from studies conducted in the American continent, the average prevalence of osteoporosis was 11.5% in women and 8.5% in men (3). This difference between sexes is due to menopause, which has hypoestrogenism as one of its main characteristics, caused by the ovarian failure characteristic of this phase (4). In addition to its role in bone metabolism, estrogen promotes the secretion of calcitonin, which inhibits bone absorption, increases vitamin D3 levels, promotes intestinal calcium absorption, and regulates the sensitivity of parathyroid hormone (PTH) to calcium in the bloodstream (5).

Consumption and/or supplementation of nutrients such as proteins, calcium, phosphorus, magnesium, and vitamin D have a well-established role in maintaining bone health and preventing osteoporosis (6). In addition to these, selenium (Se) also influences bone metabolism, since its deficiency is related to a decrease in antioxidant capacity and accumulation of reactive oxygen species (ROS), with subsequent activation of osteoclasts and reduction of osteoblastic differentiation (7–9). Different human studies have evaluated the relationship between Se and bone health (10–12). In postmenopausal women, Se status was inversely associated with bone turnover and positively so with BMD (10, 11). A negative association between Se status and bone fractures in adults and the elderly has also been observed (12).

However, these studies evaluated the plasma concentration of Se, while few studies evaluated the intake of this mineral and its relationship with bone outcomes and the results still diverge. Wang et al. (13) evaluated the dietary consumption of Se by elderly men and women. They observed that individuals with lower Se consumption had a higher prevalence of osteoporosis (13). On the other hand, a study carried out with Chinese adults of both sexes found a non-linear association between Se intake and fracture risk, demonstrating that higher Se consumption increased the risk for fractures (14).

Considering the possible contribution of Se, in addition to the nutrients known to be necessary for bone health, investigating the dietary intake of Se in postmenopausal women might help to clarify its relationship with bone mass, given the heterogeneity of available results, populations, forms of assessment, and dietary availability. Thus, in this study we verified the association between dietary Se intake and BMD in postmenopausal women.

## Materials and methods

### Study design, sample size, and population

Observational, cross-sectional, analytical study of probability sampling conducted in a climacteric and osteoporosis outpatient clinic of a university hospital in Vitória, Espírito Santo, Brazil, between June 2019 and March 2020.

The sample size was based on the number of visits performed in the aforementioned clinic from January 2018 to January 2019, which was made available on a list provided by the clinic reception. Excluding duplicate visits, 342 patients were identified. For sample calculation, the Open Source Epidemiologic Statistics for Public Health (OpenEpi^®^ Version 3.01) software was used (15). A confidence interval of 95%, a margin of error of 5%, and a prevalence of osteoporosis in women over 50 years of 21.3% were considered (16), resulting in a sample of 147 women. Initially, the women were selected by simple random drawing, contacted *via* telephone, and invited to participate in the research. In case of refusal or non-compliance with the inclusion criteria of the main project, the individual in question was excluded and replaced by another person selected in a new draw. More details about the calculation and sample size have been previously published (17). A total of 140 women were evaluated; for this study, women aged ≥50 years and in menopause for at least 12 months were included and those using hormone replacement therapy and who did not have Dual Energy X-ray Absorptiometry (DXA) in medical records were excluded.

### Study variables and instruments

Interviews for data collection were carried out at the ELSA-Brasil Research Center, in Vitória, Espírito Santo, by professionals trained and qualified for this purpose.

#### Outcome variable

The main outcome of this research was BMD, obtained by Dual Energy X-ray Absorptiometry (DXA) (GE Lunar Prodigy Advance^®^), duly calibrated and using the GE Encore^®^ software, version 14.10, configured to use the National Health and Nutrition Examination Survey (18) from the femoral neck and lumbar spine (L3 and L4 positions). All densitometry tests were performed by a trained radiology technician and the result reported by a single specialist physician to avoid interobserver variation. Data were extracted from medical records and those from exams performed up to 6 months after evaluation in the study were included. The women were classified into three groups as proposed by the World Health Organization (19): (1) Normal BMD (T-score ≥ −1.0 SD), (2) Osteopenia (T-score between −1.0 and −2.5 SD), and (3) Osteoporosis (T-score ≤ −2.5 SD).

#### Exposure variable

The dietary intake of selenium and other nutrients by the participants was evaluated by the reduced version of the ELSA-Brasil Food Frequency Questionnaire (FFQ) (20). A semi-quantitative FFQ was adapted and validated for the Brazilian population (21). Participants were asked how often they consumed the food in the last 12 months, and how much they consumed at a time. To help the participant, a response card was made available with options for frequency of consumption, in addition to a kit of standardized utensils to facilitate the identification of household measures.

To quantify the nutritional composition of the foods present in the FFQ, we used the Nutrition Data System for Research^®^–NDSR software (22), which uses the Food Composition Table of the United States Department of Agriculture (USDA) as a reference. For foods that were not found in this table, the Brazilian Food Composition Table (TACO) of the State University of Campinas was used (23). After extracting the nutritional composition data from the FFQ, the adjustment for nutrients of total energy consumption was performed using the residual method proposed by Willett et al. (24). The adjustment was performed by BMD (normal, osteopenia or osteoporosis), using the average energy of each group.

The plausibility of the energy intake data was verified and evaluated by the Goldberg cutoff point (25, 26), in which the mean energy intake (EI) estimated through the FFQ is expressed as a multiple of the mean of the basal metabolic rate (BMR) of the evaluated individuals and is compared with the possible mean energy expenditure (TEE) of these individuals, which is also expressed as a multiple of the BMR. The level of physical activity used was 1.4. The average EI/BMR ratio was 1.61. A total of 64.5% (*n* = 80) of the women had energy consumption within the limits, 12.9% (*n* = 16) had underreported energy consumption, and 22.6% (*n* = 28) had overreported energy consumption, making it plausible to use all FFQs in the analyses.

#### Covariates

Sociodemographic data such as age (years) were collected; self-declared color (27) was later classified as “white” and “non-white”; education level, categorized as “no schooling,” “elementary school,” “high school,” and “university education”; marital status, categorized as “with a partner” and “without a partner”; and employment status, categorized as “employed” and “unemployed.” Regarding lifestyle habits, alcohol consumption (“consume,” “do not consume”), smoking (“smoker,” “non-smoker”), and the physical activity (PA) level, which was obtained by the International Physical Activity Questionnaire (IPAQ), were evaluated (28). To avoid overestimating the PA level, only the sum of issues related to leisure and transportation was taken into account. Women who reported performing at least 150 mins of PA per week were classified as “sufficiently active”, while those who reported doing <150 mins of PA per week were classified as “insufficiently active,” using the recommendation of the “WHO Guidelines for Physical Activity and Sedentary Behavior” (29).

Clinical data regarding the time since menopause were self-reported and obtained from the participant's current age minus the age at which menopause was established, and presented in years. Information on the use of calcium and vitamin D supplements and drugs that affect bone metabolism was collected from medical records and categorized into “uses” and “does not use.” To assess the nutritional status, height (m) and body mass (kg) were collected according to the recommended technique (30). From these variables, the body mass index (BMI) (kg/m^2^) was calculated by dividing body mass by height squared (30). Women up to 59 years of age were classified according to the WHO (31), while women aged ≥60 years were classified according to the Pan American Health Organization (PAHO) (32).

### Ethical aspects

Individuals participated voluntarily and provided written consent by signing the Free and Informed Consent Term, after having had the research read and clarified to them, so that they were aware of the study, guaranteeing their anonymity and the confidentiality of the information obtained. This study was approved by the Research Ethics Committee of the Federal University of Espírito Santo under protocol number: 2,621,794.

### Statistical analysis

The sample was characterized through the distribution of frequencies and estimation of measures of central tendency and dispersion. The normality of the variables was assessed using the Kolmogorov-Smirnov test. The ANOVA and Kruskal-Wallis tests were applied to verify the difference between means according to data normality, while the Chi-Square and Fisher's Exact test were applied to verify the difference between proportions. The *post-hoc* Tukey and Bonferroni tests were applied to assess statistical differences between groups of parametric and non-parametric variables, respectively.

Dietary Se consumption was classified into four quartiles according to the population's own quartiles: 1st quartile (≤ 31 μg/day), 2nd quartile (32–62 μg/day), 3rd quartile (63–93 μg /day), and 4th quartile (≥94 μg/day). Odds ratios (OR) and their respective confidence intervals (CI) were calculated taking the 1st quartile as a reference. For the multivariate analysis, three adjustment models were made: 1st model: adjusted for protein (g/d), calcium (mg/d), phosphorus (mg/d), and vitamin D (μg/d) consumption; 2nd model added to model 1: age, BMI, time since menopause (continuous), physical activity level, alcohol consumption, and smoking; Model 3 added to model 2 the use of calcium and vitamin D supplements and drugs that affect bone metabolism. Data were analyzed using SPSS^®^ version 22.0 software and the significance level adopted for all tests was 5.0%.

## Results

**Figure 1:**
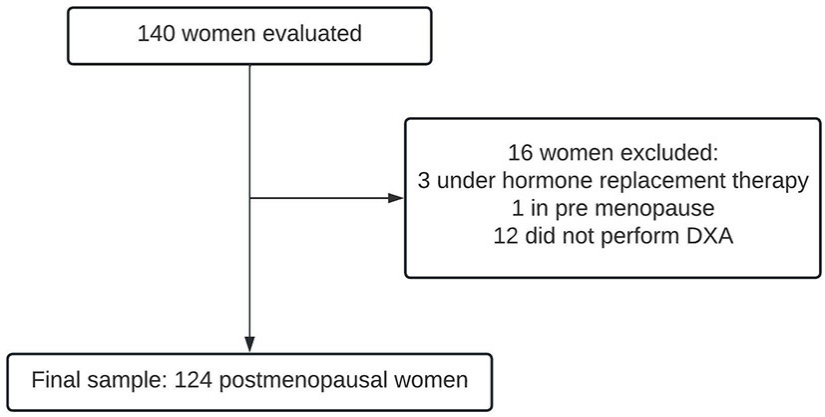
Sample selection flowchart.

The final sample consisted of 124 women (Figure 1). Sixteen women were excluded: one because she was in pre-menopause, three because they were under hormone replacement therapy, and 12 because they did not undergo DXA.

Sociodemographic and lifestyle characteristics of these women are also presented according to the Se quartile. Their mean age was 66.8 ± 6.1 years, with a predominance of women between the ages of 60 and 69.9 years (59.7%), non-white (62.1%), living with a partner (51.6%), with a basic level of schooling (60.5%), and unemployed (83.1%). As for lifestyle habits, half of the sample was classified as insufficiently active (50.0%) and the other half as sufficiently active (50.0%). Regarding alcohol consumption and smoking, most did not consume alcohol (86.3%) and did not smoke (95.2%). There was no significant difference between the quartiles of Se consumption and the variables analyzed (Table 1).

**Table 1:** Distribution of sociodemographic and lifestyle variables according to the quartiles of dietary selenium intake (μg/day) of postmenopausal women.

	**Quartiles of dietary selenium intake (**μ**g/day)**					
**Variables**	**Total (*n* = 124)**					***p*-value**
		**Q1 (≤ 31.0)**	**Q2 (32.0–62.0)**	**Q3 (63.0–93.0)**	**Q4 (≥94.0)**	
	**Mean** ±**SD**					
**Age (years)**	66.8 ± 6.1	68.0 ± 6.3	66.8 ± 6.7	65.6 ± 6.6	66.8 ± 4.7	0.492
	***n*** **(%)**					
**Age group (years)** ^a^						0.895
50.0–59.9	13 (10.5)	2 (15.4)	4 (30.8)	4 (30.8)	3 (23.1)	
60.0–69.9	74 (59.7)	18 (24.3)	17 (23.0)	18 (24.3)	21 (28.4)	
≥ 70.0	37 (29.8)	11 (29.8)	10 (27.0)	9 (24.3)	7 (18.9)	
**Color** ^b^						0.945
White	47 (37.9)	13 (27.7)	12 (25.5)	11 (23.4)	11 (23.4)	
Non-white	77 (62.1)	18 (23.3)	19 (24.7)	20 (26.0)	20 (26.0)	
**Marital status** ^b^						0.731
No partner	60 (48.4)	17 (28.3)	13 (21.7)	16 (26.7)	14 (23.3)	
With partner	64 (51.6)	14 (21.9)	18 (28.1)	15 (23.4)	17 (26.6)	
**Educational level** ^a^						0.197
No schooling	11 (8.9)	4 (36.4)	4 (36.4)	2 (18.1)	1 (9.1)	
Elementary school	75 (60.4)	21 (28.0)	20 (26.6)	17 (22.7)	17 (22.7)	
High school	27 (21.8)	5 (18.5)	6 (22.2)	10 (37.1)	6 (22.2)	
University education	11 (8.9)	1 (9.1)	1 (9.1)	2 (18.2)	7 (63.6)	
**Employment status** ^b^						0.482
Employed	21 (16.9)	4 (19.0)	4 (19.0)	8 (38.1)	5 (23.9)	
Unemployed	103 (83.1)	27 (26.2)	27 (26.2)	23 (22.3)	26 (25.3)	
**Physical activity level** ^b^						0.943
Insufficiently active	62 (50.0)	14 (22.6)	16 (25.8)	16 (25.8)	16 (25.8)	
Sufficiently active	62 (50.0)	17 (27.4)	15 (24.2)	15 (24.2)	15 (24.2)	
**Smoking** ^a^						0.631
Smoker	6 (4.8)	1 (16.7)	2 (33.3)	2 (33.3)	1 (16.7)	
Non-smoker	118 (95.2)	30 (25.4)	29 (24.6)	29 (24.6)	30 (25.4)	
**Alcohol consume** ^a^						0.794
Consume	17 (13.7)	4 (23.5)	6 (35.3)	2 (11.8)	5 (29.4)	
Do not consume	107 (86.3)	27 (25.2)	25 (23.4)	29 (27.1)	26 (24.3)	

^*a*^ *Fisher's Exact;* ^*b*^ *Chi-Square. ANOVA test, post-hoc Tukey. SD, standard deviation. Q1: 1st quartile; Q2: 2nd quartile; Q3: 3rd quartile; Q4: 4th quartile*.

Table 2 shows the distribution of clinical variables and dietary intake according to Se intake quartiles. The women in this study had a mean time since menopause of 19.6 ± 8.8 years and a mean BMI of 27.3 ± 4.7 kg/m^2^. Most of them were classified as having normal weight (42.7%), taking calcium (65.3%) and vitamin D (57.3%) supplements, and not using drugs that affect bone metabolism (53.2%). There was no significant difference between these variables according to the quartiles of Se intake.

**Table 2:** Distribution of clinical and nutrients intake variables according to the quartiles of dietary selenium intake (μg/day) in postmenopausal women.

	**Quartiles of dietary selenium intake (**μ**g/day)**					
**Variables**	**Total (*n* = 124)**					***p-*value**
		**Q1 (≤31.0)**	**Q2 (32.0–62.0)**	**Q3 (63.0–93.0)**	**Q4 (≥94.0)**	
	**Mean** ±**SD**					
**Time since menopause (Years)**	19.6 ± 8.8	22.1 ± 8.6	19.1 ± 8.5	17.6 ± 9.8	19.9 ± 8.2	0.239
**BMI (kg/m** ^2^ **)**	27.3 ± 4.7	26.0 ± 4.5	27.3 ± 4.5	28.1 ± 5.1	27.6 ± 4.6	0.351
**Energy (Kcal/d)**	2013.1 ± 791.4	1801.5 ± 1073.8	1965.9 ± 638.6	1956.8 ± 723.7	2149.2 ± 676.4	0.749
**Protein (g/d)**	87.6 ± 18.1	74.6^a^ ± 12.4	85.9^b^ ± 13.4	96.5^b^ ± 16.5	93.5^b^ ± 20.9	**<0.001**
**Selenium (μg/d)**	154.4 ± 88.7	93.4^a^ ± 15.7	118.9^b^ ± 5.1	142.1^c^ ± 10.0	263.1^d^ ± 119.9	**<0.001**
**Calcium (mg/d)**	742.4 ± 288.5	606.6^a^ ± 201.3	848.8^b^ ± 399.5	679.0^a, b^ ± 190.1	835.2^b^ ± 247.7	**0.001**
**Phosphorus (mg/d)**	1230.9 ± 249.3	1073.1^a^ ± 170.6	1238.4^b^ ± 282.3	1242.2^b^ ± 169.3	1370.0^b^ ± 267.2	**<0.001**
**Vitamin D (μg/d)**	11.5 ± 10.4	6.7^a^ ± 5.4	10.2^a, b^ ± 5.5	12.3^a, b^ ± 9.4	16.7^b^ ± 15.6	**0.019**
	***n*** **(%)**					
**Nutritional status** ^**a**^						0.230
Underweight	21 (16.9)	9 (42.9)	5 (23.8)	3 (14.3)	4 (19.0)	
Normal weight	53 (42.8)	11 (20.8)	13 (24.5)	15 (28.3)	14 (26.4)	
Overweight	18 (14.5)	6 (33.3)	5 (27.8)	1 (5.6)	6 (33.3)	
Obesity	32 (25.8)	5 (15.6)	8 (25.0)	12 (37.5)	7 (21.9)	
**Ca supplementation** ^**b**^						0.283
Yes	81 (65.3)	21 (25.9)	23 (28.4)	16 (19.8)	21 (25.9)	
No	43 (34.7)	10 (23.3)	8 (18.6)	15 (34.8)	10 (23.3)	
**Vit. D supplementation** ^**b**^						0.764
Yes	71 (57.3)	18 (25.4)	20 (28.2)	17 (23.9)	16 (22.5)	
No	53 (42.7)	13 (24.5)	11 (20.8)	14 (26.4)	15 (28.3)	
**Drugs that affect bone metabolism** ^**b**^						0.437
Yes	58 (46.8)	17 (29.3)	16 (27.6)	14 (24.1)	11 (19.0)	
No	66 (53.2)	14 (21.2)	15 (22.7)	17 (25.8)	20 (30.3)	
**BMD** ^**b**^						**0.006**
Normal	27 (21.8)	1 (3.7)	4 (14.8)	8 (29.6)	14 (51.9)	
Osteopenia	52 (41.9)	15 (28.8)	15 (28.8)	13 (25.0)	9 (17.4)	
Osteoporosis	45 (36.3)	15 (33.3)	12 (26.7)	10 (22.2)	8 (17.8)	

^*a*^ *Fisher's Exact;* ^*b*^ *Chi-Square. ANOVA post-hoc Tukey. Kruskal-Wallis Test, post-hoc Bonferroni. SD: standard deviation. P-values in bold: p* < *0.05. Q1: 1st quartile; Q2: 2nd quartile; Q3: 3rd quartile; Q4: 4th quartile. Different superscript letters indicate a significant difference (p* < *0.05) between groups. BMI, Body Mass Index; BMD, Bone Mineral Density; Ca, Calcium; Vit. D, Vitamin D*.

When assessing nutrient intake, the mean Se intake was 154.4 ± 88.7 (51.7–564.9) μg/day among the women evaluated. Those with higher consumption of Se (Q4) consumed higher levels of protein (93.5 ± 20.9 g/d; *p* < 0.001), calcium (835.2 ± 247.7 mg/d; *p* = 0.001), phosphorus (1,370.0 ± 267.2 mg/d; *p* < 0.001), and vitamin D (16.7 ± 15.6 μg/d; *p* = 0.019) compared with women with lower consumption (Q1). The energy intake did not vary between the quartiles (*p* = 0.749) (Table 2).

Regarding BMD classification, 41.9% of women were classified as having osteopenia and 36.3% osteoporosis. According to the BMD classification, 51.9% of women with normal BMD had a higher dietary intake of Se (Q4). On the other hand, 57.7% of the women with osteopenia and 60% of those with osteoporosis were among the quartiles with the lowest consumption of Se (Q1 and Q2) (*p* = 0.003). Nutritional status, calcium and vitamin D supplementation, and use of drugs that affect bone metabolism did not vary between the quartiles (*p* > 0.05) (Table 2).

Table 3 presents the association between dietary Se intake quartiles and BMD classification from multivariate logistic regression. In the crude model, the OR for osteopenia were 0.11 (95%CI: 0.01–0.99; *p* = 0.048) and 0.04 (95%CI: 0.01–0.38; *p* = 0.005) and for osteoporosis 0.08 (95%CI: 0.01–0.77; *p* = 0.029) for the third and fourth quartiles, respectively, when compared with the first quartile. After adjusting for possible confounding variables, the association between selenium intake and BMD remained negative only for osteoporosis. The OR for osteoporosis in postmenopausal women in the fourth quartile was 0.02 (95%CI: 0.001–0.41; *p* = 0.012), compared with women in the first quartile.

**Table 3:** Multivariate regression between the quartiles of dietary selenium intake (μg/day) and bone mineral density groups of postmenopausal women.

**Quartiles of dietary Se intake (μg/day)**	**Osteopenia**				**Osteoporosis**			
	**Crude model**	**Model 1**	**Model 2**	**Model 3**	**Crude model**	**Model 1**	**Model 2**	**Model 3**
	**OR (CI 95%)**	**OR (CI 95%)**	**OR (CI 95%)**	**OR (CI 95%)**	**OR (CI 95%)**	**OR (CI 95%)**	**OR (CI 95%)**	**OR (CI 95%)**
**Q2 (32.0–62.0)**	**0.25 (0.03–2.51)**	**0.50 (0.04–6.22)**	**0.81 (0.05–12.89)**	**1.01 (0.06–16.77)**	**0.20 (0.02–2.03)**	**0.20 (0.02–2.51)**	**0.34 (0.02–6.01)**	**0.36 (0.02–7.37)**
**Q3 (63.0–93.0)**	**0.11 (0.01–0.99)**	**0.13 (0.01–1.45)**	**0.32 (0.02–5.02)**	**0.61 (0.03–10.72)**	**0.08 (0.01–0.77)**	**0.06 (0.01–0.76)**	**0.22 (0.01–3.97)**	**0.68 (0.03–17.40)**
**Q4 (≥ 94.0)**	**0.04 (0.01–0.38)**	**0.10 (0.01–1.08)**	**0.09 (0.01–1.22)**	**0.08 (0.01–1.18)**	**0.03 (0.004–0.35)**	**0.04 (0.003–0.42)**	**0.03 (0.002–0.55)**	**0.02 (0.001–0.41)**

*Reference group: 1st Quartile and Normal bone mineral density; OR: Odds Ratio; CI 95%: Confidence Interval of 95%. Q2: 2nd quartile; Q3: 3rd quartile; Q4: 4th quartile. Values in bold: p< 0.05*.

*Model 1: adjusted for protein, calcium, phosphorus and vitamin D*.

*Model 2: adjusted for protein, calcium, phosphorus, vitamin D, age, body mass index, time since menopause, physical activity level, alcohol consumption and smoking*.

*Model 3: adjusted for protein, calcium, phosphorus, vitamin D, age, body mass index, time since menopause, physical activity level, alcohol consumption, smoking, calcium and vitamin D supplementation, and drugs that affect bone metabolism*.

## Discussion

The present study revealed an inverse association between Se consumption and the presence of osteoporosis. Women who were in the highest Se consumption quartile were less likely to have osteoporosis compared with those in the lowest quartile of Se consumption.

Few studies have evaluated dietary Se consumption and its outcomes in relation to BMD and osteoporosis. In line with our results, Wang et al. (13), when evaluating 6,267 Chinese adults aged in average 52.2 years, of which 2,640 were women, found a negative association between Se intake assessed from an FFQ and the prevalence of osteoporosis in women [0.53 (95%CI: 0.32–0.89), *p* = 0.018], even after adjusting for age, BMI, energy intake, smoking, alcohol consumption, physical activity level, fiber intake, calcium, dietary supplements, diabetes, and hypertension (13). However, BMD was evaluated in the medial phalanges of the non-dominant hand by compact radiographic absorptiometry, a method that is not considered the gold standard (19).

Wolf et al. (33) evaluated 11,068 women between 50 and 79 years old and did not find an association between Se consumption and BMD; however, most women had normal BMD, which may have caused the loss of association (33). De França et al. (34), when evaluating antioxidant intake and BMD in Brazilian postmenopausal women, also found no association between Se intake and BMD at various bone sites (femoral neck, total femur, lumbar spine, and whole-body) (34). Pedrera-Zamorano et al. (35) found that high Se consumption negatively affected BMD in postmenopausal women aged over 51 years only if calcium intake was below 800 mg/d. When calcium consumption exceeded 800 mg/d, Se intake appeared to no longer affect BMD (35). These results contradict those obtained in this study, which showed the high consumption of Se as a protective factor for the development of osteoporosis.

The average consumption of Se by the women evaluated in the present study was 154.4 ± 88.7 μg/d, which is above the 55 μg/d recommended for women over 50 years of age by the Dietary Reference Intakes (DRI) (36) and the averages reported in similar studies (85.9, 43.5 and 95.5 μg/d) (13, 33, 35), although it was similar to that of a study with Brazilian postmenopausal women (108 μg/d) (34). One of the hypotheses for the high intake of Se in our study may be related to the eating habits of the study population. The women evaluated reported consuming food sources such as meat, eggs, and nuts both frequently and in high amounts. Se can be obtained from food sources such as seafood, meat, eggs, and cereals. Brazil nuts have approximately 95.5 μg of Se in just one unit (5 g) (37), which may explain our results. When evaluating the consumption of antioxidants in 2,344 Brazilian adults, Pinheiro et al. (38) found that women consumed an average of 84.8 ± 40.1 μg/d of Se, and only 11.5% of women had low consumption of this mineral (38), demonstrating that the consumption of Se by the Brazilian population is higher than the nutritional recommendations (36). Still, women had higher dietary intake of protein, calcium, phosphorus, and vitamin D. This finding can be explained by the consumption of foods that are sources of these nutrients, since meat, chicken, fish, and eggs are foods rich in proteins and contain high levels of selenium, and milk and dairy products also contribute a considerable fraction of dietary intake of Se (39).

Evidence indicates that Se is involved in bone remodeling and metabolism, and consequent decreased BMD (10–13). It is known that bone metabolism is controlled by RANKL, an NF-κB ligand, present on the surface of osteoblasts. RANKL binds to its receptor, RANK, expressed on the surface of osteoclasts, and stimulates osteoclast activity and bone resorption. An exacerbated resorption will cause bone loss that cannot be overcome by bone formation (9). Se modulates this process through several mechanisms: the anti-inflammatory property presented by this mineral reduces the expression of NF-κB and inhibits the activity of interleukin-6 (IL-6) and other inflammatory cytokines (40), thus regulating bone turnover, since these cytokines mediate the maturation of osteoclasts and the increase in bone resorption (41, 42). Additionally, Se can stimulate mitochondrial biogenesis, playing a protective role in mitochondrial function and preventing metabolic bone damage, as it induces apoptosis in mature osteoclasts through mitochondrial pathways (43). Furthermore, Se has an important antioxidant role through its selenoproteins, suppressing the generation of ROS and inhibiting osteoclast differentiation by supressing RANKL (9, 44, 45). Finally, the selenoenzymes iodothyronine deiodinases protect the thyroid hormones from oxidative stress and regulate blood circulation. A Se deficiency can lead to an excess of these hormones in the blood, consequently increasing bone turnover and accelerating bone loss, which results in osteoporosis (46, 47). Therefore, inadequate Se intake can alter bone metabolism by decreasing the redox capacity, thus increasing ROS formation and inducing oxidative stress and inflammation in an unregulated way. This exacerbation increases bone resorption and compromises bone microarchitecture (9, 45).

This study has several strengths. First, BMD was obtained from DXA of the lumbar spine (L2–L4) and femoral neck, the gold standard method for the diagnosis of osteoporosis (19). Second, women on hormone replacement therapy were excluded. Third, by adjusting the multivariate model for several potential confounders, the reliability of the results was strengthened. However, some limitations are present. Because of the cross-sectional design of this study, it is not possible to state a causal relationship between Se consumption and the development of osteoporosis, suggesting that longitudinal studies, especially in this population, should be performed. The quantification of Se from the FFQ can be impaired, as the Se content in foods can vary depending on the region (48) and due to inherent flaws in the evaluation method (49), although the FFQ used was validated for the Brazilian population (21). The use of glucocorticoids was not evaluated and women who used drugs that affect bone metabolism such as bisphosphonates were not excluded from the analyses, although that was used as an adjustment in the multivariate model. Also, data on sun exposure and outdoor activities, which are important for vitamin D synthesis and bone health, were not collected.

In conclusion, Se consumption was associated with BMD in postmenopausal women, and those with higher Se consumption were less likely to have osteoporosis. Based on this premise, our results expand the knowledge about the relationship between Se and the maintenance of bone health, although longitudinal investigations are necessary to clarify the role of Se in BMD and its consequences, since the results are still conflicting (10–14, 33–35).

## Data availability statement

The raw data supporting the conclusions of this article will be made available by the authors, without undue reservation.

## Ethics statement

The studies involving human participants were reviewed and approved by Research Ethics Committee of the Federal University of Espírito Santo. The patients/participants provided their written informed consent to participate in this study.

## Author contributions

PG, GC, BA, JM-R, TP, and VG contributed to conception and design of the study. PG, CV, and GC conducted research. PG, TP, and VG performed the statistical analysis, wrote the paper, and had primary responsibility for final content. All authors contributed to manuscript revision, read and approved the submitted version.

## Conflict of interest

The authors declare that the research was conducted in the absence of any commercial or financial relationships that could be construed as a potential conflict of interest.

## Publisher's note

All claims expressed in this article are solely those of the authors and do not necessarily represent those of their affiliated organizations, or those of the publisher, the editors and the reviewers. Any product that may be evaluated in this article, or claim that may be made by its manufacturer, is not guaranteed or endorsed by the publisher.
